# Nitric Oxide: Renoprotective in Cardiac Surgery!

**DOI:** 10.21470/1678-9741-2020-0080

**Published:** 2020

**Authors:** Rohan Magoon, Souvik Dey, Ashish Walian, Ramesh Kashav

**Affiliations:** 1 Department of Cardiac Anaesthesia, Atal Bihari Vajpayee Institute of Medical Sciences (ABVIMS) and Dr. Ram Manohar Lohia Hospital, Baba Kharak Singh Marg, New Delhi, India. E-mail: rohanmagoon21@gmail.com; 1 Department of Cardiac Anaesthesia, Atal Bihari Vajpayee Institute of Medical Sciences (ABVIMS) and Dr. Ram Manohar Lohia Hospital, Baba Kharak Singh Marg, New Delhi, India.; 1 Department of Cardiac Anaesthesia, Atal Bihari Vajpayee Institute of Medical Sciences (ABVIMS) and Dr. Ram Manohar Lohia Hospital, Baba Kharak Singh Marg, New Delhi, India.; 1 Department of Cardiac Anaesthesia, Atal Bihari Vajpayee Institute of Medical Sciences (ABVIMS) and Dr. Ram Manohar Lohia Hospital, Baba Kharak Singh Marg, New Delhi, India.

**To the Editor,**

The impact of nitric oxide (NO) on renal function in critically ill patients is controversial. A broadly inclusive meta-analysis by Ruan et al. demonstrated a worsened renal function with NO therapy in acute respiratory distress syndrome (ARDS) patients whereas no clinically significant effect was outlined in non-ARDS subset, signifying that the effect of NO on kidney might be specific to the underlying pathophysiology^[1]^.

It is noteworthy that two studies included in the aforementioned meta-analysis were contemplated in cardiac surgical patients, wherein post-cardiopulmonary bypass (CPB) NO administration did not incur an elevated risk of acute kidney injury (AKI)^[1,2]^. Interestingly, Lei et al. were the first group to delineate a renoprotective potential of NO in their randomized controlled trial (RCT) on 244 patients undergoing valve replacement surgeries. A peculiar characteristic of their study was the administration of 80 parts per million NO (via gas exchanger) on CPB followed by an inhalational route for the first 48 postoperative hours. The NO group was characterized by a reduced incidence of postoperative AKI and any major adverse kidney event at the first year following surgery compared to the control group, which received nitrogen^[3]^.

A recent meta-analysis of five RCTs evaluating the effect of NO on renal function after cardiac surgeries performed on CPB revealed intriguing results in the subgroup analysis, highlighting a lower incidence of AKI only with the NO therapy initiated from the beginning of CPB^[2]^. On the other hand, post-CPB NO therapy did not result in a reduced AKI incidence, pointing towards plausible attenuated renal insult during the extracorporeal circulatory assistance.

A close look at the mechanisms of AKI in cardiac surgery provides clues to the novel observation. While a conglomeration of factors, such as microcirculatory dysfunction, tissue hypoperfusion, inflammatory milieu, ischemia-reperfusion injury, and hemodilution, predispose to renal insult, hemolysis (particularly with a prolonged CPB) is a major factor in aggravating the underlying injury. Hemolysis in turn leads to decreased plasma NO bioavailability, culminating as a diminished tissue perfusion owing to the absence of vasodilatory effects of NO^[4]^. In addition, plasma haemoglobin accumulates as a consequence of excessive hemolysis, which further scavenges NO and accentuates the glomerular injury. A range of animal and human physiological studies have elucidated the role of exogenous NO in oxidising the ferrous plasma oxyhemoglobin to ferric methemoglobin (MetHb), augmenting an enhanced plasma NO bioavailability during the extracorporeal circulatory support^[5]^. However, if NO is administered following CPB, the hemolysis sequel already sets in and the NO-associated improvement in right ventricular function and the consequently relieved venous congestion are the only probable mechanisms in limiting CPB-induced renal injury.

Despite the interesting results of the meta-analysis, a number of caveats need to be considered while comprehending the significance in the clinical context. Firstly, the small number of eligible RCTs enrolled and the heterogeneous AKI definition in the included RCTs interrogate the generalization potential of the inferences. Moreover, a trial sequential analysis of three RCTs employing Kidney Disease: Improving Global Outcomes (KDIGO) criteria failed to demonstrate a definite conclusion, necessitating future studies^[2]^. Secondly, the degree of NO-associated methemoglobinemia merits additional evaluation, which is pertinent in a setting predisposed to AKI, albeit the finding of a clinically insignificant MetHb elevation with NO therapy on CPB emanating from the index meta-analysis^[2]^. Lastly, the logistic and cost issues of NO therapy compound the situation.

To conclude, the aforementioned discussion of the renoprotective potential of NO in cardiac surgery constitutes an important endeavour towards the goal of ameliorating AKI in this vulnerable patient cohort. The results of an on-going phase III clinical trial at the Massachusetts General Hospital evaluating the renoprotective role of NO in cardiac surgical population with endothelial dysfunction are ardently anticipated in order to further align the preliminary observations in this area of clinical interest^[6]^.
